# Fusion of Bilateral Lower-Limb Neuromechanical Signals Improves Prediction of Locomotor Activities

**DOI:** 10.3389/frobt.2018.00078

**Published:** 2018-06-26

**Authors:** Blair Hu, Elliott Rouse, Levi Hargrove

**Affiliations:** ^1^Center for Bionic Medicine, Shirley Ryan AbilityLab, Chicago, IL, United States; ^2^Department of Biomedical Engineering, Northwestern University, Evanston, IL, United States; ^3^Department of Mechanical Engineering, University of Michigan, Ann Arbor, MI, United States; ^4^Department of Physical Medicine and Rehabilitation, Northwestern University, Chicago, IL, United States

**Keywords:** intent recognition, sensor fusion, assistive devices, bilateral, locomotion

## Abstract

Wearable lower-limb assistive devices have the potential to dramatically improve the walking ability of millions of individuals with gait impairments. However, most control systems for these devices do not enable smooth transitions between locomotor activities because they cannot continuously predict the user's intended movements. Intent recognition is an alternative control strategy that uses patterns of signals detected before movement completion to predict future states. This strategy has already enabled amputees to walk and transition seamlessly and intuitively between activities (e.g., level ground, stairs, ramps) using control signals from mechanical sensors embedded in the prosthesis and muscles of their residual limb. Walking requires interlimb coordination because the leading and trailing legs have distinct biomechanical functions. For unilaterally-impaired individuals, these differences tend to be amplified because they develop asymmetric gait patterns; however, state-of-the-art intent recognition approaches have not been systematically applied to bilateral neuromechanical control signals. The purpose of this study was to determine the effect of including contralateral side signals for control in an intent recognition framework. First, we conducted an offline analysis using signals from bilateral lower-limb electromyography (EMG) and joint and limb kinematics recorded from 10 able-bodied subjects as they freely transitioned between level ground, stairs, and ramps without an assistive device. We hypothesized that including information from the contralateral side would reduce classification errors. Compared to ipsilateral sensors only, bilateral sensor fusion significantly reduced error rates; moreover, only one additional sensor from the contralateral side was needed to achieve a significant reduction in error rates. To the best of our knowledge, this is the first study to systematically investigate using simultaneously recorded bilateral lower-limb neuromechanical signals for intent recognition. These results provide a device-agnostic benchmark for intent recognition with bilateral neuromechanical signals and suggest that bilateral sensor fusion can be a simple but effective modular strategy for enhancing the control of lower-limb assistive devices. Finally, we provide preliminary offline results from one above-knee amputee walking with a powered leg prosthesis as a proof-of-concept for the generalizability and benefit of using bilateral sensor fusion to control an assistive device for an impaired population.

## Introduction

Worldwide, millions of individuals experience conditions such as stroke, spinal cord injury, and limb loss, which can cause severe and lasting gait impairments that limit functional independence and reduce quality of life (Verghese et al., 2006). Recent advances in mechatronic design and embedded systems have also led to the proliferation of wearable assistive devices that can provide locomotion assistance by actuating lower-limb joints. Such devices include robotic lower-limb prostheses, orthoses, and exoskeletons (e.g., Varol et al., 2010; Quintero et al., 2012; Mooney et al., 2014; Ottobock, 2015, 2016; Panizzolo et al., 2016; Young and Ferris, 2017). Compared to their mechanically passive counterparts, powered devices can be controlled to actively change their mechanical properties between different locomotor activities (e.g., level ground, stairs) and to inject energy into the system (e.g., powered plantarflexion in late stance). However, to maximize the potential benefits of powered assistance and to avoid disrupting the gait cycle, these devices must predict state changes before they occur. Currently, though, most control systems for these assistive devices require the user to explicitly indicate an intended transition with a key fob or an unnatural pre-programmed motion pattern (e.g., bouncing up and down on the Ottobock C-Leg) (Ottobock, 2015). Although the human-machine control interface varies among lower-limb assistive devices, their control systems share similar ideals. To restore normal walking ability, they should accurately infer and execute the user's locomotor intent in a manner that is automatic, seamless, and intuitive to the user.

To more intuitively infer the user's locomotor intent for control, intent recognition has been successfully developed for and primarily applied to powered lower-limb prostheses as an alternative strategy for predicting the appropriate assistance to provide. We define the intent recognition control framework as using information from the human, assistive device, and/or environment detected before movement completion (e.g., windows extracted before heel contact or toe off events) to predict the user's upcoming locomotor activity on a step-by-step basis (Varol et al., 2010). Several studies have already demonstrated the benefits of *unilateral sensor fusion for controlling a prosthesis with intent recognition strategies which can operate in real-time on embedded systems*. For example, neuromechanical sensor fusion of EMG from the residual limb and prosthesis load information from five above-knee amputees walking with a passive device significantly reduced error rates compared to either sensor set alone (Huang et al., 2011). Fusing above-knee EMG with a diverse set of mechanical sensors embedded in a powered knee-ankle prosthesis comprised of potentiometers and encoders at the knee and ankle, an axial load cell, and 6-degree-of-freedom (DOF) inertial measurement unit (IMU) on the shank also significantly reduced error rates (Young et al., 2014a). In subsequent work, the addition of a 6-DOF load cell and calculated thigh and shank inclination angles to the existing set of mechanical sensor information further reduced error rates; the control system also continued to benefit from fusion with EMG (Spanias et al., 2015). As an alternative to EMG, capacitive sensing has also been used for intent recognition with below-knee amputees (Zheng et al., 2014).

In addition to these unilateral sensor fusion strategies, other powered prosthesis-specific control system modifications (e.g., merging ramp ascent and level walking classes, using mode-specific classifiers, and delaying predictions by 90 ms) have further reduced error rates (Hargrove et al., 2015; Simon et al., 2017; Spanias et al., 2018). Error rates during online sessions (i.e., the user interacts dynamically with the control system) using state-of-the-art intent recognition strategies have approached approximately 4% (Spanias et al., 2018); although impressive, they must be further reduced before intent recognition can be used to control a powered assistive device safely and reliably over long periods of time. Despite promising potential for controlling powered prostheses, intent recognition is still not commonly applied to controlling devices for individuals with impaired but intact limbs. Notably, powered orthoses and exoskeletons differ from prostheses because they assist by supplementing instead of substituting the movement of the instrumented limb(s). A few devices have used multimodal sensor fusion for control but they typically rely on pre-defined thresholds to switch between locomotor activities and are mostly limited to identifying transitions between sitting, standing, and level ground walking. For instance, the estimated location of the center of pressure controls switching between sitting, standing, and walking modes of a powered hip-knee orthosis for paraplegic individuals (Quintero et al., 2011, 2012). Ground reaction forces, posture, EMG, and electroencephalography (EEG) have also been used to infer user intent in order to synchronize robotic assistance with paraplegic subjects' movement during gait initiation/termination and level ground walking (Suzuki et al., 2007; Kilicarslan et al., 2013). Bilateral lower-limb neuromechanical signals have also been used to predict sitting, standing, and walking in one patient with multiple sclerosis using intent recognition (Zhang and Huang, 2013). Yet regardless of the devices or control signals used, incorrectly predicting locomotor activities still presents challenges for the long-term clinical viability of intent recognition.

Prediction errors can be categorized as steady-state or transitional, depending on whether the true locomotor activities before and after each gait event are the same (i.e., steady-state) or different (i.e., transitional). Whereas transitional errors can be especially destabilizing and more likely to result in injury (e.g., at the top of the stairs when descending) steady-state errors are harder to anticipate and more frustrating for users. Although intent recognition algorithms can produce seamless transitions during online use, transitional error rates for prosthesis control are still much higher than for steady-state steps (Hargrove et al., 2015; Spanias et al., 2015, 2018; Simon et al., 2017). Walking, especially transitions, requires bilateral coordination of the lower body. For example, the anticipatory lower-limb joint mechanics and EMG signals for able-bodied subjects differed for transitions from level ground walking to stair ascent and descent and for leading and trailing legs (Peng et al., 2016). The mechanical work performed by each leg also differs for both uphill and downhill walking (Franz et al., 2011). Although individuals with unilateral gait impairment typically develop new patterns of interlimb coordination, their non-affected limb generally remains anatomically and biomechanically intact (Chen et al., 2005; Segal et al., 2006; Ingraham et al., 2016). Yet, nearly all assistive devices that are commercially available and/or used in research settings do not incorporate information from both legs and it remains unknown whether contralateral side signals contain rich and robust enough information about the user's intent to justify their inclusion. For example, the powered Vanderbilt knee-ankle prosthesis (Varol et al., 2010) and hydraulic C-Leg knee prosthesis (Ottobock, 2015) and C-Brace knee ankle foot orthosis (Ottobock, 2016) are all controlled using only signals from the affected side; however, we hypothesized that information from the unaffected leg could improve controllability.

Previously, instrumenting the unaffected leg was impractical and considered a major barrier to clinical feasibility. Also, approaches for incorporating information from the contralateral limb have been limited beyond echo control (Grimes et al., 1977; Grimes, 1979; Joshi et al., 2010) and complementary limb motion estimation (Vallery et al., 2011). Echo control required cyclical activities, for which movement had to be initiated by the unaffected side, because the kinematic trajectory of the intact limb was simply “replayed” on the prosthesis side with a half-step delay. Complementary limb motion estimation, which uses residual body motion and interjoint couplings to infer an appropriate reference trajectory for the impaired limb(s), is a more intuitive and cooperative control strategy but has only been implemented for position control. Now, minimally invasive wearable sensors capturing neuromechanical signals are becoming more ubiquitous and can be more easily placed on the contralateral limb to supplement control information from sensors embedded in an assistive device. For instance, soft bio-electronics for physiological recording are already clinically viable (Liu et al., 2016). With these recent developments, minimally invasive bilateral instrumentation of the lower extremities is becoming more feasible. But to our knowledge, only a few studies have investigated *bilateral sensor fusion for intent recognition*. For example, able-bodied subjects wore bilateral pressure insoles and unilateral IMU sensors on the thigh, shank, and foot (Chen et al., 2014) or walked in a lower-limb exoskeleton with embedded sensors measuring bilateral ground reaction forces and shank/foot orientation to control knee assistance (Long et al., 2016). Although both studies achieved low error rates, their findings may not translate well to seamless, online control because they used prediction periods spanning the entire upcoming step instead of only the instant when the upcoming step begins.

Therefore, we still lack a clear understanding of both how bilateral sensor fusion across different modalities systematically affects intent recognition error rates and whether prosthesis-derived intent recognition strategies perform well when generalized to non-prosthesis applications. In this study we present a proof-of-concept for an intent recognition control system using a broad set of bilateral lower-limb neuromechanical signals recorded from wearable sensors instrumented on able-bodied subjects freely walking without an assistive device. Our overall objective was to conduct an offline analysis to systematically compare and benchmark the performance of unilaterally and bilaterally -informed intent recognition control systems and to identify the most critical sensors. We confirmed our hypothesis that sensor fusion across different modalities and across legs would reduce steady-state and transitional error rates. We also report preliminary results from a separate offline analysis on one unilateral above-knee amputee walking with a powered knee-ankle prosthesis to demonstrate the benefit of incorporating kinematic information from the unimpaired leg to improve control of an assistive device with intent recognition. We expect our positive results to further the ongoing development of and broaden the scope of intent recognition strategies for controlling wearable lower-limb assistive devices.

## Materials and methods

### Experimental protocol

This study was carried out in accordance with the recommendations of the Northwestern University Institutional Review Board with written informed consent from all subjects. Following IRB approval, 10 able-bodied subjects (7 male, 3 female; 23–29 years, 160–193 cm, 54–95 kg) completed the experiment. Before walking, subjects were instrumented bilaterally with wearable sensors to measure lower limb muscle activity and joint and limb kinematics. EMG signals were recorded using bipolar surface electrodes (DE2.1; Delsys, Boston, MA, USA) from the same seven muscles in each leg: tibialis anterior (TA), medial gastrocnemius (MG), soleus (SOL), vastus lateralis (VL), rectus femoris (RF), biceps femoris (BF), and semitendinosus (ST). These muscles were chosen because they are in part responsible for hip and knee flexion/extension and ankle plantarflexion/dorsiflexion, movements that are commonly assisted by wearable devices. They are also relatively easy to target when facing the subject from in front and behind and are similar to muscle sites used by Sylos-Labini et al. (2014). Electrode placement was guided by the Surface ElectroMyoGraphy for the Non-Invasive Assessment of Muscles (SENIAM, seniam.org) standards. We palpated to locate the muscle belly and oriented the electrode along the primary fiber direction (Kendall et al., 2005), and verified placement by having subjects perform maximum voluntary contractions. The muscle sites were prepared by removing excess hair and the skin was cleaned by mildly scrubbing with an alcohol wipe. Sensors were attached to the skin with a double-sided adhesive. Signals were amplified by 1000x, hardware band-pass filtered between 20 and 450 Hz (Bagnoli 16, Delsys), and sampled at 1 kHz.

Joint kinematic signals (sagittal plane only) were recorded using electrogoniometers (SG150; Biometrics Ltd, Newport, UK) placed along the knee and ankle and sampled at 500 Hz. 6-DOF (tri-axial accelerometer and gyroscope) IMU's were placed bilaterally on the subjects' thigh (below RF EMG electrode) and shank (adjacent to TA EMG electrode) and sampled at 500 Hz (MPU-9250; Invensense, San Jose, CA, USA). All signals were simultaneously recorded with a custom 16-bit data acquisition device that permits multi-rate sampling. We chose these wearable sensors because they are analogous to sensors commonly embedded in prostheses, orthoses, and exoskeletons (such as joint position encoders and shank/thigh IMU's) and are more easily integrated with existing device-based sensorization in a hypothetical hybrid system. Other force and interaction torque sensors such as load cells and strain gauges were excluded because they are not as relevant for our device-agnostic approach and are more difficult to integrate if not already embedded in the device.

**Figure 1:**
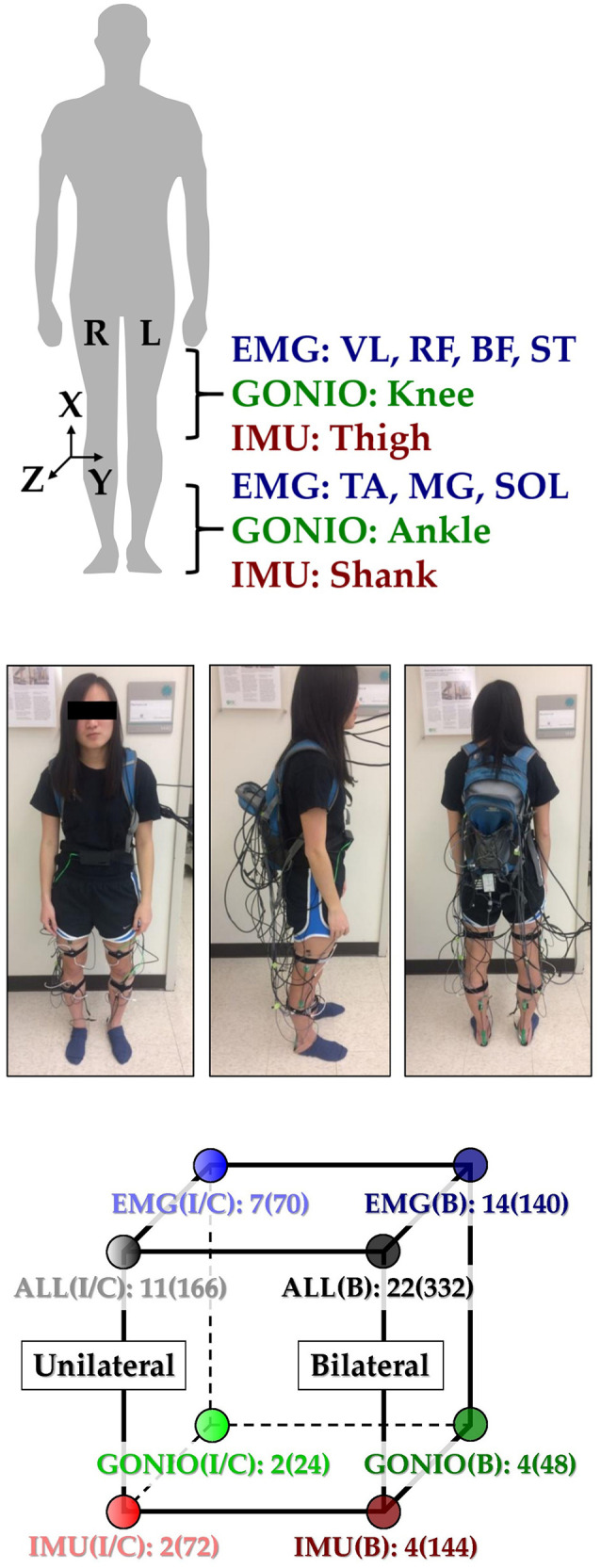
Instrumentation setup showing EMG, goniometer, and IMU sensor placement [adapted from Hu et al. (2018)]. **(Top)** 11 total sensors (labeled) were placed on each leg. The coordinate frame of IMU sensors is also shown. **(Middle)** A representative subject instrumented with all bilateral sensors in a tethered setup. The subject provided written informed consent for the publication of this image. **(Bottom)** Classifier comparisons for four modality groups and three laterality groups: EMG, goniometer (GONIO), IMU, fused (ALL), ipsilateral (I), contralateral (C), and bilateral (B). The number of sensors and extracted features (in parentheses) are shown next to each classifier configuration.

To facilitate integration with our data acquisition software, all wearable sensors were used in a tethered setup; as a drawback, fully instrumenting one leg took up to an hour. The full instrumentation setup is shown for a representative subject in (Figure 1 top, middle). Prior to data collection, the goniometers were calibrated while the subject was in the upright standing position. In an experimental session, each subject completed approximately 25 repetitions of a circuit consisting of walking on level ground (LW), ascending/descending a ramp with a 10° slope (RA/RD), and ascending/descending a four-step staircase (SA/SD) step-over-step using a data collection procedure previously described in Young et al. (2014b). These activities were chosen because they encompass the main types of terrain likely encountered in community ambulation. Subjects were instructed to freely transition between activity modes at their self-selected speed while the experimenter labeled the true locomotor intent of the subject using a key fob.

### Signal processing

Heel contact and toe off gait events for each leg were reliably identified by finding peaks in the low-pass filtered (1st order Butterworth, 6 Hz) sagittal plane angular velocity of the shank segment using a dual-minima method similar to (Jasiewicz et al., 2006; Maqbool et al., 2016). Briefly, the largest peaks in angular velocity were first used to identify mid-swing events. Toe off and heel contact events were identified by searching for peaks before and after each mid-swing event, respectively. Gait initiation and termination strides and trials during which the subject paused, stumbled, or tripped were excluded. For each subject, there were 530 ± 46 heel contact events and 536 ± 45 toe off events for each leg (mean ± standard deviation). EMG signals were first high-pass filtered (6th order Butterworth) at 20 Hz to attenuate motion artifact (De Luca et al., 2010). EMG signals were then notch-filtered (6th order Butterworth, 6 Hz width) at 60, 180, and 300 Hz (based on spectral analysis) to remove ambient interference. Goniometer and IMU signals were low-pass filtered (6th order Butterworth) at 10 and 25 Hz, respectively. Because we did not use foot-mounted IMU's, joint velocities could not be estimated using inertial signals only for the ankle. For consistency, we indirectly estimated joint velocities for both the knee and ankle by taking the centered-difference numerical derivative of the low-pass filtered joint position signals instead and added these velocities to the goniometer channels.

All signals were segmented into 300 ms analysis windows before each identified heel contact or toe off gait event (one window/event). For each window, we extracted features previously used in intent recognition for online control of a powered knee-ankle prosthesis. Features for goniometer and IMU signals included the mean, standard deviation, maximum, minimum, initial, and final values (6 features/channel) (Varol et al., 2010). Features for EMG signals included the mean absolute value (MAV), waveform length, number of zero crossings and slope sign changes, and the coefficients of a sixth-order autoregressive model (10 features/channel) (Huang et al., 2005; Hargrove et al., 2008). These heuristic features were chosen because they can be computed efficiently on an embedded system and concisely capture the general shape of mechanical signals and the frequency content of EMG signals. Bilaterally, there were a total of 22 sensors (14 EMG, 4 goniometer, 4 IMU) and 46 channels (14 EMG, 8 goniometer, 24 IMU). The feature dimensionality for all ipsilateral and bilateral signals was 166 and 332, respectively (Figure 1, bottom).

### Offline classifier evaluation

**Figure 2:**
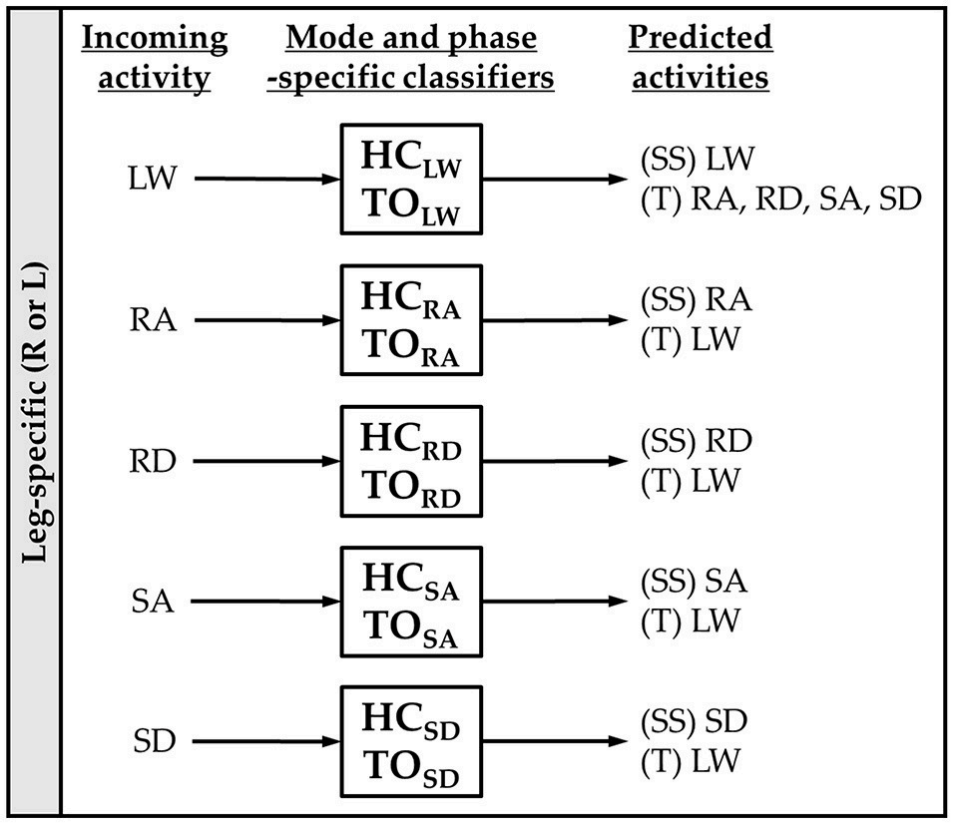
Mode-specific classification scheme. Ten classifiers were trained for each leg corresponding to all combinations of incoming activity [level walking (LW), ramp ascent (RA), ramp descent (RD), stair ascent (SA), and stair descent (SD)] and gait event [heel contact (HC) and toe off (TO)]. The possible predictions for each classifier are listed as steady-state (SS) or transitional (T) activities.

For each subject, we evaluated leg- and mode/phase-specific classifiers (e.g., right heel contact, left toe off) for several sensor sets to compare their offline error rates (Figure 2). We used a mode-specific classification scheme previously developed for powered leg prosthesis control (Young and Hargrove, 2016), which achieves lower error rates by encoding domain knowledge about the allowable transition(s) from each mode. Briefly, 20 total classifiers were trained to encompass all combinations of the four gait events (right/left heel contact or toe off) and five locomotor activities (level ground, ramp ascent/descent, stair ascent/descent). The total number of steps used to train each classifier (combined between legs for all subjects) is reported in Table 1. The appropriate classifier for each prediction was selected based on the activity just before the gait event (i.e., incoming activity based on the key fob label). The possible outputs for each classifier (i.e., predicted activities) only included remaining in the current locomotor activity or transitioning to another allowable mode (e.g., in stair ascent mode, remaining in stair ascent or transitioning back to level walking but excluding stair descent and ramp ascent/descent). The error rate was defined as the proportion of incorrectly classified gait events in the testing set and was computed by averaging across legs and gait events for each subject. Errors were also categorized as steady-state or transitional. We performed randomized 10-fold cross-validation for each subject for 12 different sensor sets (Figure 1, bottom) using the steps collected from all circuits completed during the experimental session. The ipsilateral side was defined as the side on which the gait event was identified, which could have been either the leading or trailing leg. Sensors were divided into four modality groups: EMG only (EMG), goniometer only (GONIO), IMU only (IMU), or all combined (ALL). Sensors were also divided into three laterality groups: ipsilateral (I), contralateral (C), or bilateral (B).

**Table 1:** Number of training examples for each mode-specific classifier.

**Classifier**	***LW***	***RA***	***RD***	***SA***	***SD***	**Total**
HC_LW_	4,523	240	240	239	248	5,490
TO_LW_	4,637	245	246	253	243	5,624
HC_RA_	243	1,408				1,651
TO_RA_	252	1,416				1,668
HC_RD_	239		1,757			1,996
TO_RD_	245		1,762			2,007
HC_SA_	238			489		727
TO_SA_	245			472		717
HC_SD_	248				475	723
TO_SD_	242				478	720

*The correct labels are listed in the header and instances are aggregated across subjects and between legs*.

Linear discriminant analysis (LDA) has emerged as a convenient *a priori* choice of classifier for intent recognition for the control of upper- and lower-limb prostheses because it provides a good compromise between classification accuracy and computational efficiency (Hargrove et al., 2007; Scheme and Englehart, 2011). Other commonly used classifiers include support vector machines (SVM) and artificial neural networks (ANN), which can represent more complex, non-linear decision boundaries and may be more appropriate for modeling transitions. The sensor set containing all bilateral signals contains more features than previously used in intent recognition for lower-limb prostheses so we also assessed the effect of classifier type on error rates for ipsilateral and bilateral sensor sets containing all modalities. For all classifiers, the feature data were normalized to have zero mean and unit variance. For LDA, the input dimensionality was further reduced using principal components analysis (PCA) to preserve 95% of the total variance and the prior for each classifier was set to be equiprobable. Hyperparameters for SVM (one-vs-one, linear kernel, *C* = 10 using the scikit-learn Python package) and ANN (one hidden layer with 10 units, hyperbolic tan activation function, stochastic gradient descent with momentum, adaptive learning rate initialized to 0.1 using the scikit-learn Python package) were chosen based on pilot data.

We performed repeated measures ANOVA for LDA classifiers with error rate as the response variable, modality and laterality (ipsilateral and bilateral only) as fixed within-subject factors, and subject as a random factor. We expected some modalities would benefit more from bilateral information so we included an interaction term. Post-hoc comparisons (paired *t*-test) with Bonferroni correction were conducted on statistically significant factors. We also used paired *t-*tests to compare ipsilateral and contralateral sensor sets. We performed repeated measures ANOVA for the combined sensor set with error rate as the response variable, laterality and classifier as fixed within-subject factors, and subject as a random factor. We expected more complex classifiers to perform worse for higher dimensionality data so we included an interaction term. Post-hoc comparisons (paired *t*-test) with Bonferroni correction were conducted on statistically significant factors.

### Optimal sensor selection

To determine the optimal number and type of sensors to instrument on the contralateral leg, we performed sequential forward selection for each subject to choose the sensors which minimized overall error rate with LDA classification (10-fold cross-validation), beginning with all ipsilateral sensors as the baseline and ending with all bilateral sensors. We chose not to identify the bilaterally optimal sensor combination (i.e., beginning with the empty set) because we were primarily interested in the effect of adding contralateral sensors. After each iteration, all features associated with the selected sensor were added to the existing feature set and the selected sensor was removed from the set of remaining sensors. The composition of the sensor set after each iteration was recorded. We performed repeated measures ANOVA with error rate as the response variable, iteration as a fixed within-subject factor, and subject as a random factor. We also performed post-hoc comparisons (paired *t*-test) between iterations using a Bonferroni correction.

### Preliminary application to controlling a powered leg prosthesis

#### Experimental protocol

**Figure 3:**
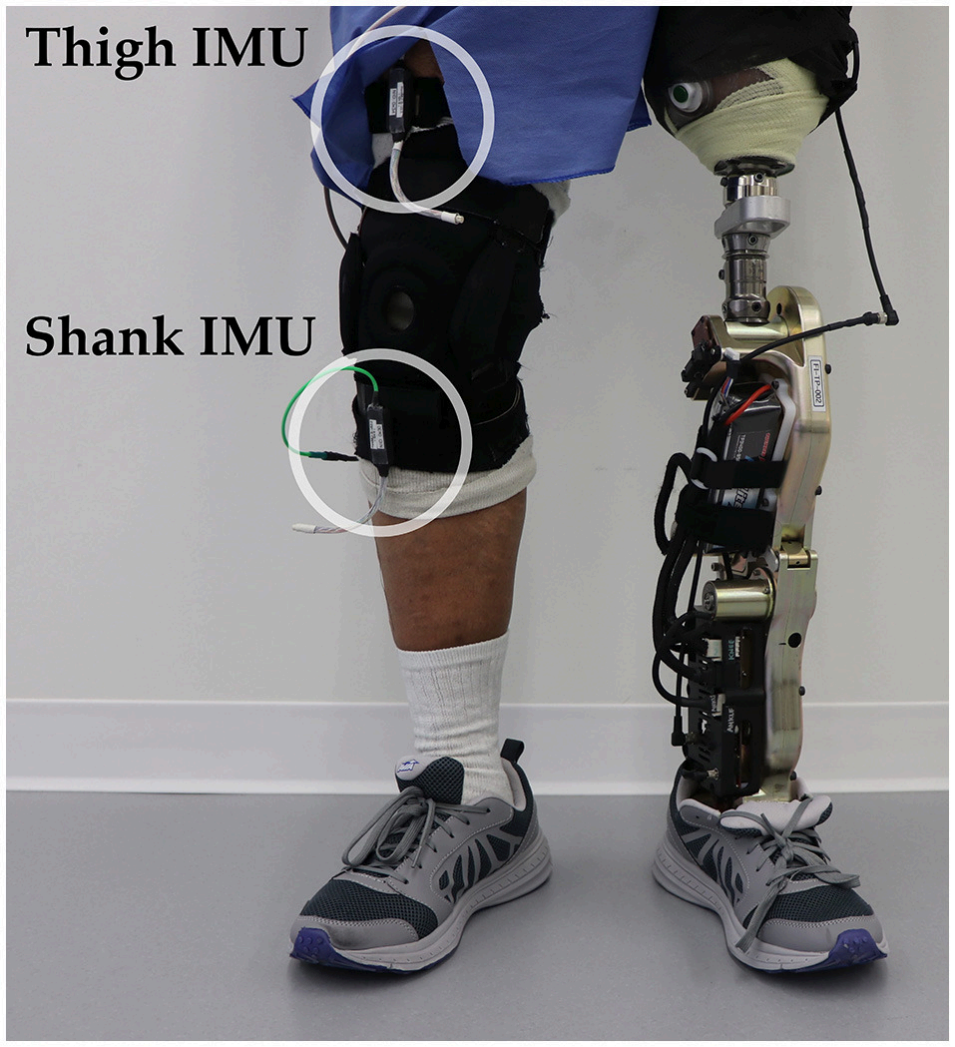
Bilateral sensorization for powered leg prosthesis control. Inertial measurement units were placed on the above-knee amputee subject's non-prosthesis side shank and thigh during offline data collection.

One individual with a left traumatic above-knee amputation (59 years, 48 years post-amputation, 83.9 kg, Medicare K3 functional level) gave written informed consent to participate in this study. The user was fitted to the Vanderbilt powered knee-ankle prosthesis (Varol et al., 2010) by a certified prosthetist and was experienced walking with the device (minimum of 5 h) (Simon et al., 2014). During data collection, the experimenter manually triggered the powered prosthesis into the correct mode as the user performed tasks including shuffling while standing and walking on level ground and ascending/descending stairs and ramps. To add variability to these movements, the subject was instructed to vary walking speed, include pauses, modify step length, use different angles of approach, and limit upper body support (e.g., only one hand on railing). The user always led with the sound side for stair ascent approaches, with the prosthesis side for stair descent approaches, and either side for ramp ascent and descent approaches. Data from 17 mechanical sensors embedded in the prosthesis were recorded at 500 Hz including knee and ankle joint position and velocity, motor currents, prosthesis acceleration and angular velocity, calculated thigh and shank inclination angles, and axial load. Two additional 6-DOF (tri-axial accelerometer and gyroscope) IMU's were worn by the subject on the non-prosthesis side thigh and shank (Figure 3) and sampled at 250 Hz (MPU-9250; Invensense, San Jose, CA, USA). The locomotor activity and state (i.e., phase of the gait cycle) of the prosthesis were also recorded to label the data.

#### Signal processing

The inclination angles of the non-prosthesis side shank and thigh were calculated using a complementary filter and added to the set of recorded signals to match inertial signals from the prosthesis side. All signals were segmented into 300 ms analysis windows around gait events (i.e., heel contact, mid-stance, toe off, mid-swing). Features including the mean, standard deviation, maximum, minimum, initial, and final values were extracted from each window (6 features/channel) (Varol et al., 2010). With the addition of both non-prosthesis side shank and thigh IMU's, the total number of channels was 31 and the feature dimensionality was 186. Feature data were normalized to have zero mean and unit variance. Consistent with previous studies (e.g., Simon et al., 2017), the dimensionality of the feature data was reduced to 50 using PCA to prevent overfitting.

#### Offline classifier evaluation

To assess the offline classification accuracy, we implemented a state-of-the-art mode-specific classification scheme which uses delayed transitions (i.e., windows started 210 ms before the gait event and ended 90 ms after the gait event) to control a powered leg prosthesis using intent recognition (same as Simon et al., 2017). This baseline classifier also merged the level ground and ramp ascent data because those activities have similar device assistance settings and previous studies have shown that combining those activities is appropriate. We compared the baseline classifier to a more generic one which neither delays transitions nor merges level walking and ramp ascent activities. We used LDA for all eight mode-specific classifiers (Simon et al., 2017). Errors were categorized as steady-state or transitional and error rate was defined as the proportion of incorrectly classified gait events in the testing set after averaging across all classifiers. We performed leave-one-out cross-validation on all the steps recorded during the experimental session for four different sensor sets: prosthesis sensors only, prosthesis with contralateral shank or thigh IMU, and prosthesis with both contralateral IMU's.

## Results

### Bilateral neuromechanical signals and features

**Figure 4:**
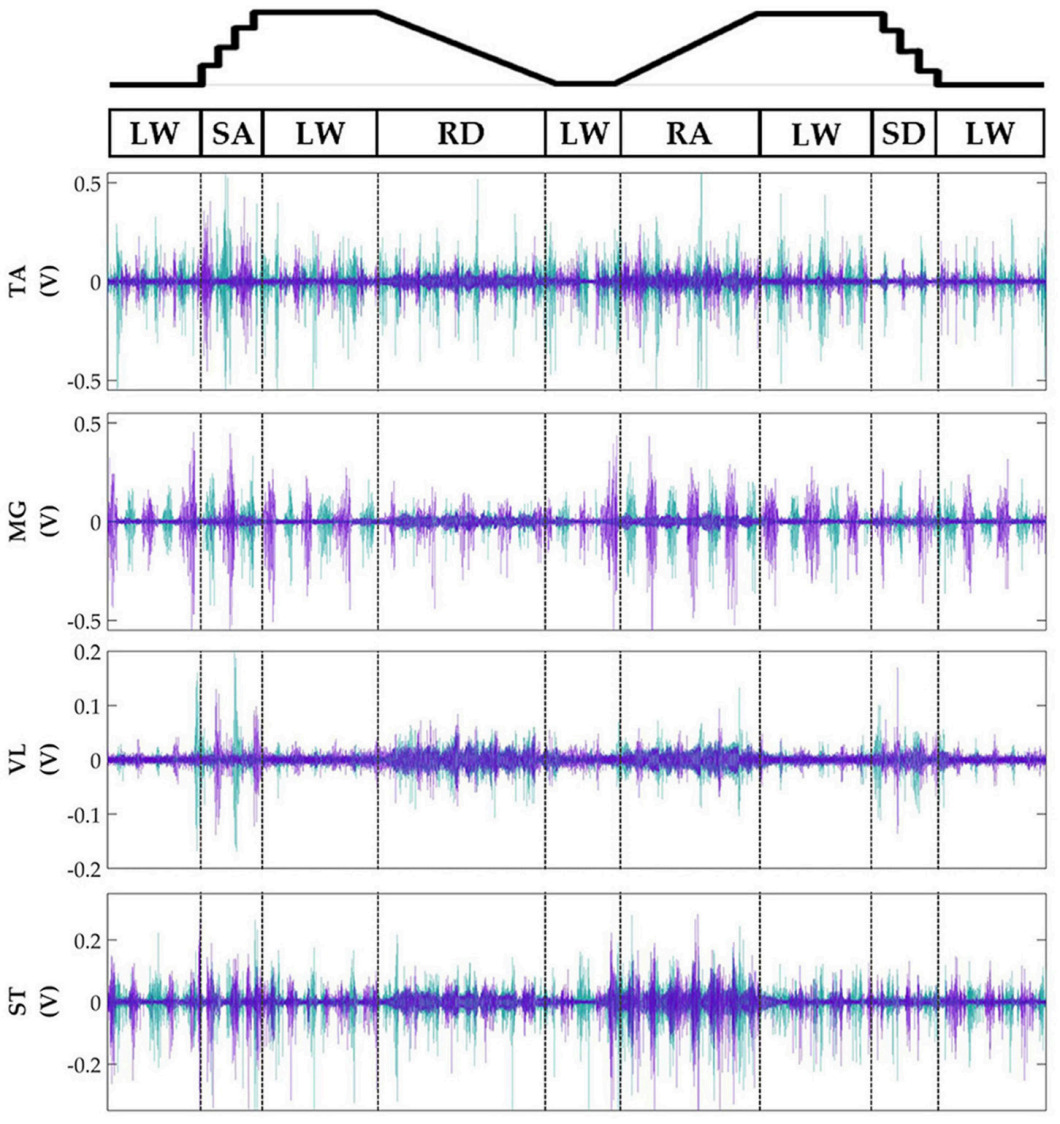
Representative bilateral post-processed EMG signals. Bilateral filtered EMG (in volts) from upper and lower leg muscles for one subject for a complete circuit consisting of level walking (LW), ramp ascent/descent (RA/RD), and stair ascent/descent (SA/SD). Turquoise traces represent the right leg and purple traces represent the left leg. Circuits were recorded as two discontinuous trials (LW→SA→LA→RD→LW and LW→RA→LW→SD→LW) but are represented as continuous.

**Figure 5:**
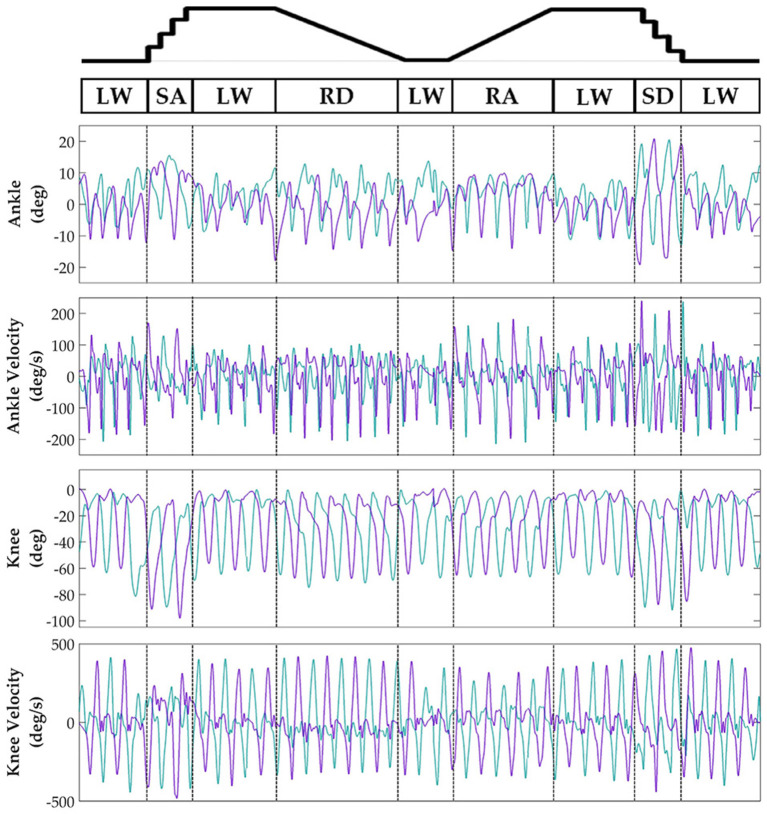
Representative bilateral post-processed joint kinematic signals. Filtered joint position (and estimated velocities) recorded from knee and ankle goniometers. Turquoise traces represent the right leg and purple traces represent the left leg. Circuits were recorded as two discontinuous trials (LW→SA→LA→RD→LW and LW→RA→LW→SD→LW) but are represented as continuous.

**Figure 6:**
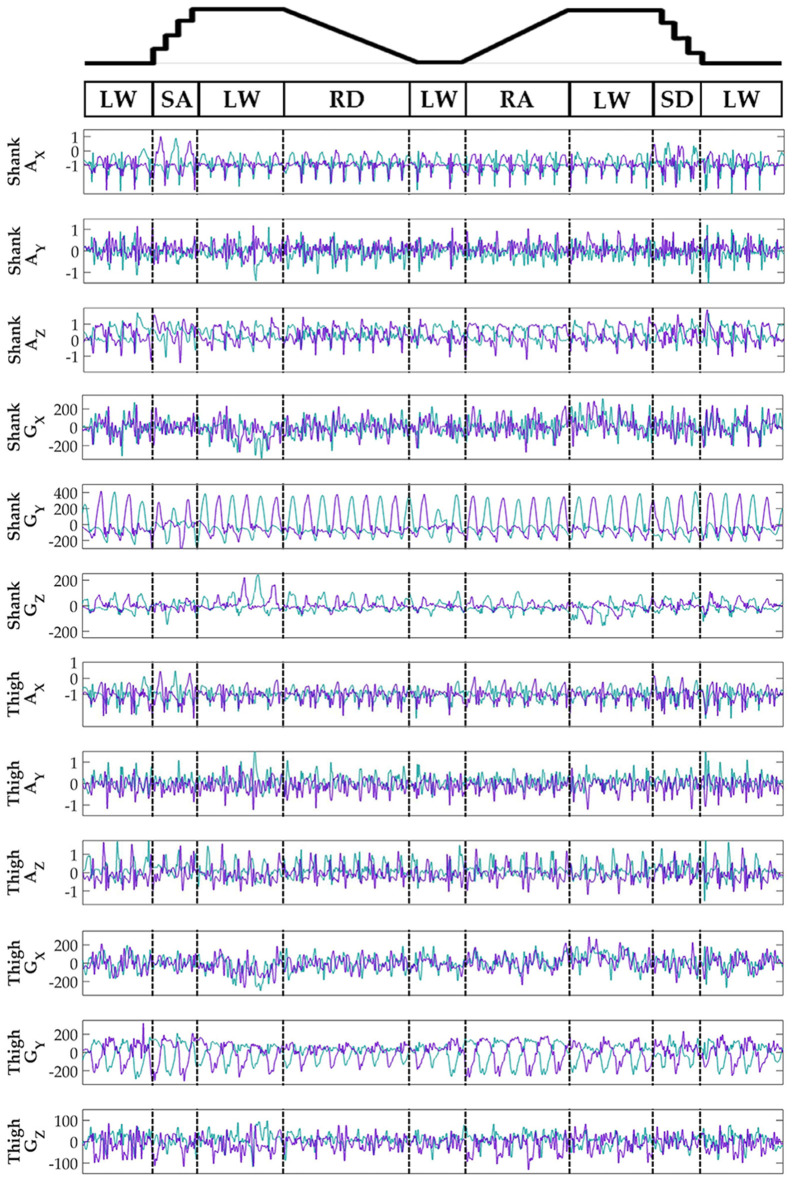
Representative bilateral post-processed limb kinematic signals. Filtered limb kinematics recorded from shank and thigh IMU's. Accelerometer (A_X_, A_Y_, A_Z_, units in g's) and gyroscope (G_X_, G_Y_, G_Z_, units in deg/s). Sagittal plane limb movement is represented in G_Y_. Turquoise traces represent the right leg and purple traces represent the left leg. Circuits were recorded as two discontinuous trials (LW→SA→LA→RD→LW and LW→RA→LW→SD→LW) but are represented as continuous.

**Figure 7:**
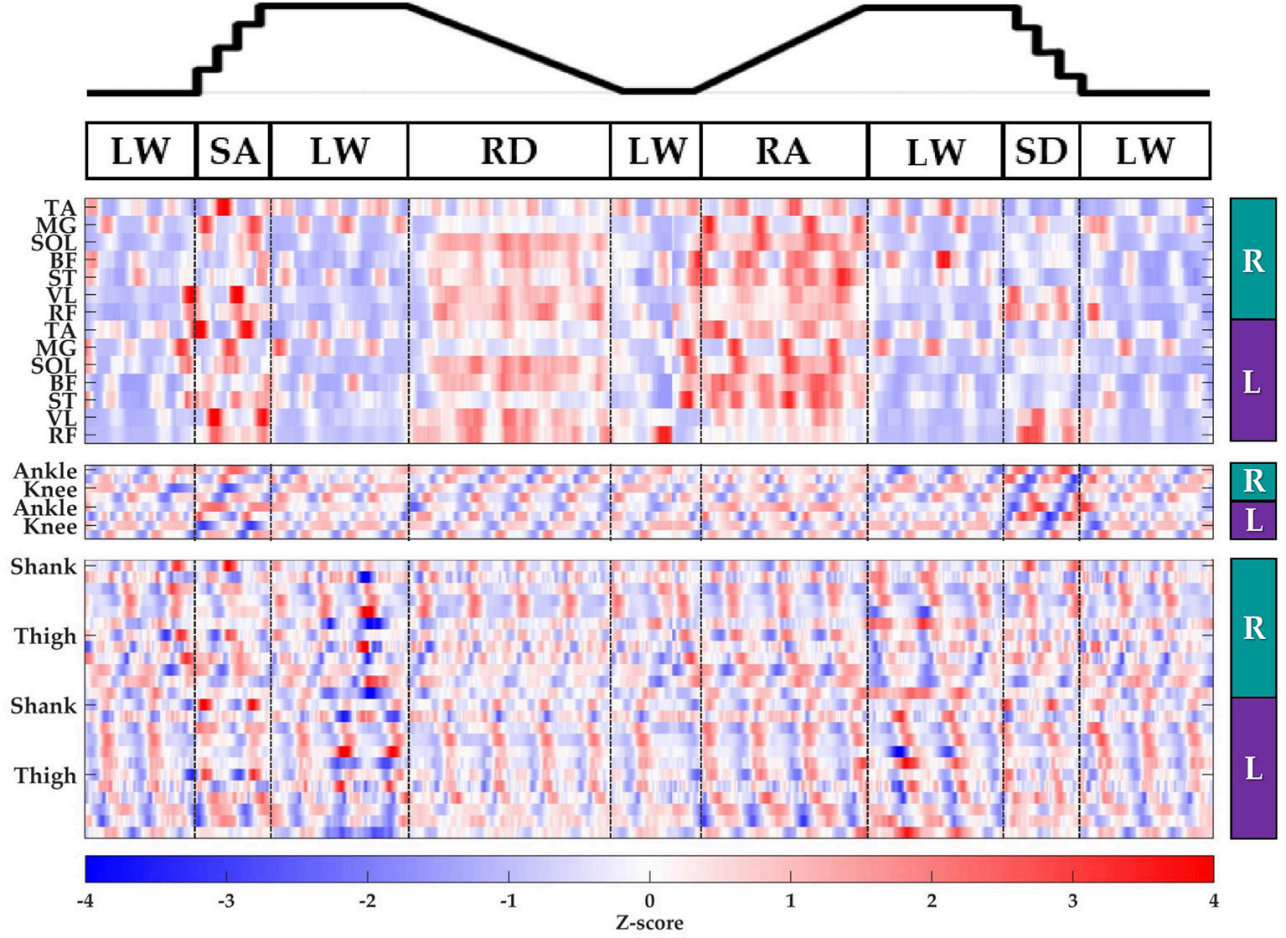
Representative raster plot of bilateral features. The mean value of each channel (row) for each leg (right, R; left, L) was extracted from sliding windows (length 300 ms, increment 30 ms) for one subject for a complete circuit consisting of level walking (LW), ramp ascent/descent (RA/RD), and stair ascent/descent (SA/SD). *Z*-scores (represented by the color bar) were computed along each row. Distinct patterns could be visually identified for many additional features (not shown).

Subjects' bilateral neuromechanical signals were distinguishable based on locomotor activity and mostly consistent between legs and trials throughout the experimental session. Representative data depicting all sensors except SOL, RF, and BF (similar to MG, VL, and ST, respectively) are shown in Figure 4 (EMG), Figure 5 (goniometer), and Figure 6 (IMU) for one representative circuit. Qualitatively, unique patterns of activation in the feature space (Figure 7) of certain channels aligned closely with different activities and their associated transitions (only mean value features shown for overlapping 300 ms windows).

### Offline classifier evaluation

**Figure 8:**
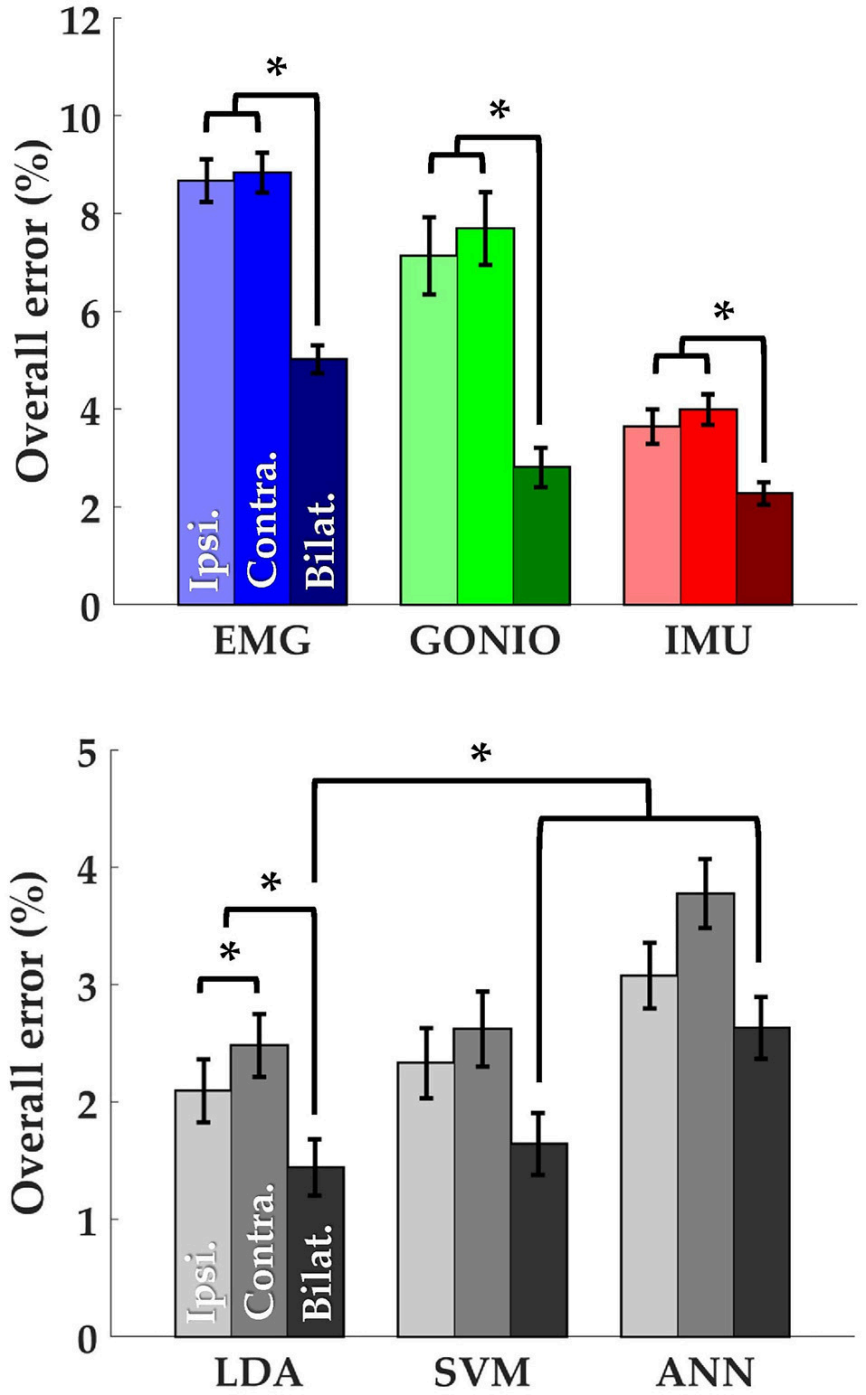
Bilateral sensor fusion reduces intent recognition errors. **(Top)** Overall error rates (mean ± SEM) for single modality sensor sets. **(Bottom)** Overall error rates (mean ± SEM) for fused sensor set for linear discriminant analysis (LDA), support vector machine (SVM), and artificial neural network (ANN) classifiers. Data are averaged across 10 subjects and each set of bars preserves the order of laterality (ipsilateral, left; contralateral, middle; bilateral, right). Asterisks denote statistically significant differences.

There was a significant interaction effect between modality and laterality (*p* = 5.53 × 10^−7^). Simple main effects analysis showed that overall error rates for classifiers using bilateral sensors were significantly reduced compared to their unilateral counterparts for almost all modality groups and machine learning algorithms (Figure 8, Table 2). There was a significant effect of classifier (*p* = 2.22 × 10^−16^) for the combined sensor sets without interaction between classifier and laterality (*p* = 0.32). Steady-state and transitional error rates were also significantly reduced when using bilateral sensors for all modality groups and machine learning algorithms with the exception of steady-state errors with SVM and ANN for the fused sensor set (ALL) (Table 2). There was generally no significant difference between unilateral (ipsilateral versus contralateral) single modality sensor sets for overall, steady-state, or transitional error rates; however, contralateral sensors had significantly higher transitional error rates with LDA and overall and transitional error rates with ANN for the fused sensor set (Table 2). For unilateral sensor sets, the error rate of IMU was lower than EMG or GONIO and decreased with sensor fusion; the lowest average overall error rate (1.43 ± 0.24%) was achieved by the LDA classifier using all bilateral sensors. The random effect of subject was significant (*p* < 10^−10^) and the overall error rate using all bilateral sensors with LDA ranged from 0.52 to 2.78%. Interestingly, there was no significant difference between overall error rates for either GONIO(B) and ALL(I) (*p* = 0.08) or IMU(B) and ALL(I) sensor sets (*p* = 0.26). For ALL(I), there was no significant difference between classifiers; however for ALL(B), the overall error rate of LDA was significantly lower than ANN (*p* = 3.02 × 10^−5^) but not different from SVM *(p* = 0.11).

**Table 2:** Complete offline classifier comparison.

	**Ipsi. (I)**	**Contra. (C)**		**Bilat. (B)**	
**OVERALL (%)**					
**EMG**	8.66 [0.44]	8.83 [0.41]	(0.51)	5.01 [0.29]*	(2.57 × 10^−7^)
**GONIO**	7.13 [0.79]	7.69 [0.74]	(0.35)	2.80 [0.41]*	(2.89 × 10^−6^)
**IMU**	3.63 [0.35]	3.98 [0.31]	(0.09)	2.26 [0.23]*	(3.43 × 10^−5^)
**ALL-LDA**	**2.09 [0.27]**	**2.48 [0.27]**	(7.19 × 10^−3^)	1.43 [0.24]*	(2.91 × 10^−6^)
**ALL-SVM**	2.33 [0.30]	2.62 [0.32]	(0.20)	1.64 [0.26]*	(1.12 × 10^−4^)
**ALL-ANN**	3.07 [0.28]	3.77 [0.29]*	(1.52 × 10^−3^)	2.63 [0.26]	(6.26 × 10^−3^)
	*α = 0.05/16		*p*-value		*p*-value
**STEADY-STATE (%)**					
**EMG**	6.61 [0.41]	6.76 [0.32]	(0.55)	3.26 [0.22]*	(1.89 × 10^−6^)
**GONIO**	6.59 [0.85]	7.16 [0.75]	(0.38)	2.26 [0.38]*	(1.21 × 10^−5^)
**IMU**	2.60 [0.31]	2.92 [0.28]	(0.13)	1.33 [0.19]*	(3.34 × 10^−5^)
**ALL-LDA**	1.25 [0.19]	1.38 [0.17]	(0.24)	0.76 [0.14]*	(6.27 × 10^−5^)
**ALL-SVM**	**1.00 [0.17]**	**1.14 [0.16]**	(0.30)	**0.71 [0.14]**	(8.95 × 10^−3^)
**ALL-ANN**	1.71 [0.16]	2.04 [0.21]	(0.02)	1.55 [0.14]	(0.15)
	*α = 0.05/12		*p*-value		*p*-value
**TRANSITIONAL (%)**					
**EMG**	17.83 [0.78]	18.18 [1.22]	(0.67)	12.91[0.85]*	(3.02 × 10^−7^)
**GONIO**	9.51 [0.82]	10.06 [0.94]	(0.46)	5.18 [0.63]*	(2.79 × 10^−6^)
**IMU**	8.31 [0.74]	8.70 [0.64]	(0.36)	6.40 [0.56]*	(6.08 × 10^−4^)
**ALL-LDA**	**5.94 [0.84]**	7.42 [0.82]*	(9.70 × 10^−4^)	4.50 [0.76]*	(5.19 × 10^−4^)
**ALL-SVM**	8.37 [1.04]	9.30 [1.22]	(0.19)	5.84 [0.89]*	(6.18 × 10^−5^)
**ALL-ANN**	9.16 [0.93]	11.52 [0.97]*	(6.94 × 10^−4^)	7.46 [1.00]*	(3.91 × 10^−3^)
	*α = 0.05/12		*p*-value		*p*-value

*Error rates (mean [SEM]) are shown for all evaluated classifiers. Asterisks under contralateral denote statistically significant differences between contralateral and ipsilateral sensor sets (p-value). Asterisks under bilateral denote statistically significant differences between bilateral and ipsilateral sensor sets (p-value). The best-performing classifier for each type of error is bolded*.

### Contralateral sensor selection

**Figure 9:**
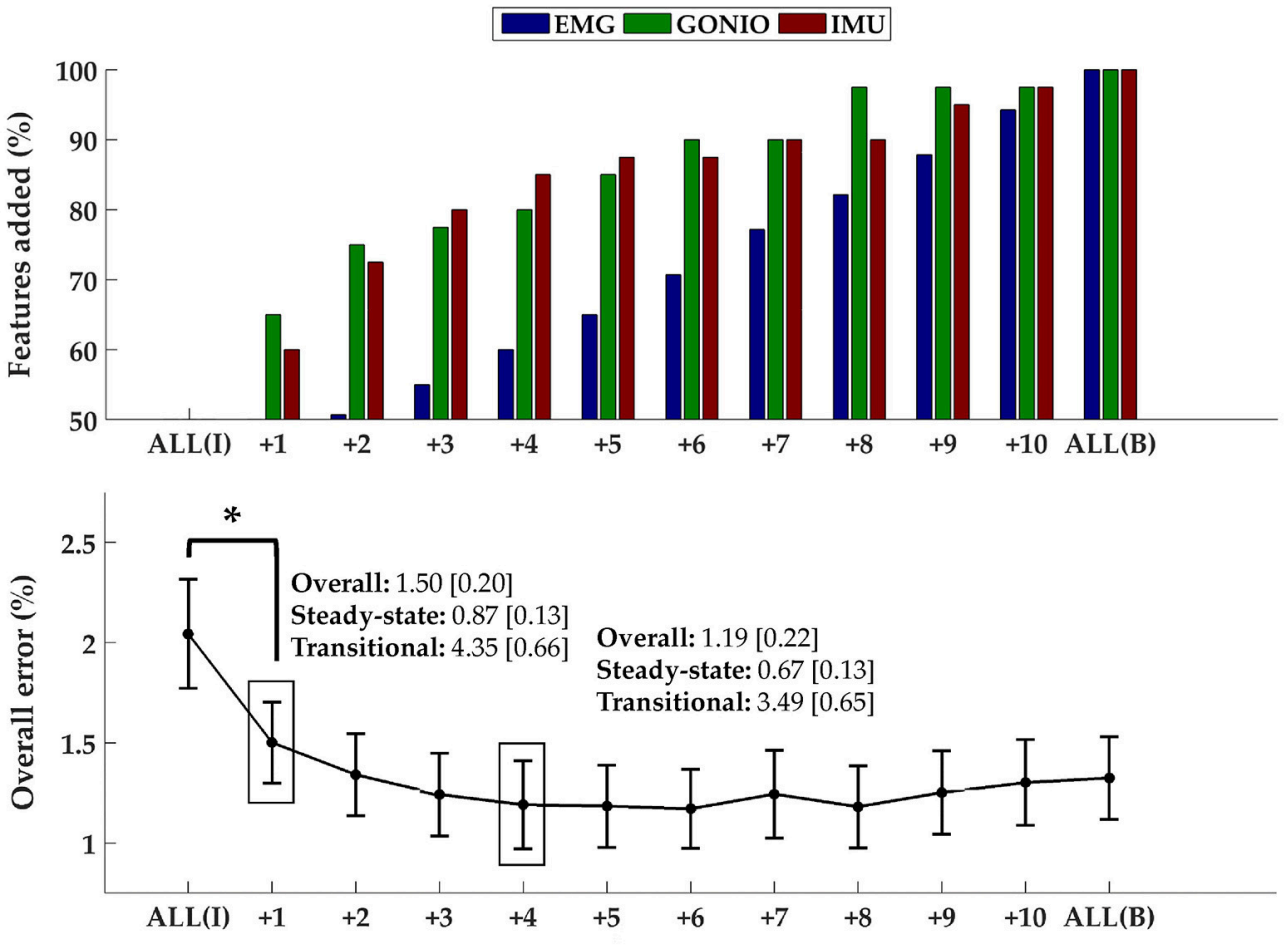
Sequential addition of contralateral sensors reduces error rates. **(Top)** The composition of the feature set was expressed as the average cumulative proportion of the total features from each modality after each iteration (displayed from left to right, most to least beneficial). **(Bottom)** The overall error rate (mean ± SEM) after each iteration. The steady-state and transitional error rates are shown after the addition of one and four contralateral sensor(s). Asterisk denotes a statistically significant difference (*p* = 1.27 × 10^−4^). Data were averaged across 10 subjects.

The optimal collection of additional contralateral sensors after each iteration varied between subjects but on average goniometer and IMU sensors were preferentially selected before EMG sensors (Figure 9, top). One contralateral lower leg kinematic sensor (i.e., ankle goniometer or shank IMU) was selected within the first two iterations for every subject; additionally, at least one goniometer sensor (i.e., ankle or knee) was selected within the first two iterations for all but two subjects. Overall (*p* = 1.27 × 10^−4^), steady-state (*p* = 2.35 × 10^−3^), and transitional (*p* = 2.54 × 10^−3^) error rates were significantly reduced from baseline after only one additional contralateral sensor (Figure 9, bottom). After four iterations, the error rate plateaued and even increased slightly when approaching all bilateral sensors.

### Preliminary application to controlling a powered leg prosthesis

Adding kinematic information from the non-prosthesis side modularly and consistently reduced offline steady-state and transitional error rates for both the baseline delayed/merged classifier and the more generic classifier (dagger symbol) (Table 3). Sensor fusion with non-prosthesis side sensors yielded the greatest relative improvement for the heel contact level walking and ramp/stair descent classifiers. For the more generic classifier which used both additional IMU's (Row 7), transitional error rates were slightly better than baseline (Row 1) but overall error rates were still higher because the toe off classifier performed worse. By using prosthesis signals only for the toe off classifier only (Row 8), the performance of the more generic classifier improves and matches the baseline classifier (Row 1).

**Table 3:** Offline error rates using bilateral sensor information to control a powered leg prosthesis with an intent recognition framework.

		**Steady-state (%)**	**Transitional (%)**	**Overall (%)**
1.	Prosthesis only	0.53	3.75	0.90
2.	Prosthesis, Contra Shank	0.36	3.00	0.67
3.	Prosthesis, Contra Shank^†^	1.26	4.99	1.69
4.	Prosthesis, Contra Thigh	0.30	2.50	0.55
5.	Prosthesis, Contra Thigh^†^	1.42	3.99	1.72
6.	Prosthesis, Contra Thigh/Shank	0.20	1.50	0.35
7.	Prosthesis, Contra Thigh/Shank^†^	1.02	3.49	1.31
8.	Prosthesis, Contra Thigh/Shank ▾	0.53	3.74	0.90
	Total decisions	3,027	400	3,427

Error rates are shown for different mode-specific classifier configurations with varying amounts of kinematic information from the non-prosthesis side. The total number of classifier decisions for each step type is also shown in the bottom row.

† *Control system neither merges LW and RA classes nor includes 90 ms delay*.

▾ *Control system neither merges LW and RA classes nor includes 90 ms delay; toe off classifier uses prosthesis signals only*.

## Discussion

In this study, we simultaneously recorded bilateral lower-limb neuromechanical (EMG, goniometer, IMU) signals from able-bodied subjects as they spontaneously transitioned between locomotor activities without wearing an assistive device. We applied a previously implemented mode-specific intent recognition framework (Young and Hargrove, 2016) to these signals for offline classification. Our primary objective was to determine the effect of including control information from the contralateral limb on offline classification accuracy. We found that using all bilateral signals achieved significantly lower overall (1.43 ± 0.24%, 32% reduction), steady-state (0.76 ± 0.14%, 39% reduction) and transitional (4.50 ± 0.76%, 24% reduction) error rates compared to all ipsilateral signals, which were generally not different from contralateral signals. There was a large inter-subject range for the lowest achievable error rates but using bilateral information consistently and significantly reduced offline error rates even for single modality sensor sets. Sequential forward selection identified an optimal subset of contralateral sensors that performed as well as, if not better than all bilateral sensors. When compared to all ipsilateral sensors as the baseline, adding only one contralateral sensor (preferentially goniometer or IMU) significantly reduced overall, steady-state, and transitional error rates. We also demonstrated in a proof-of-concept offline analysis the potential for modularly incorporating kinematic information from the non-prosthesis side to improve an amputee's control of a powered leg prosthesis with intent recognition. Placing two additional IMU sensors on the non-prosthesis side shank and thigh reduced overall, steady-state, and transitional error rates for the state-of-the-art classifier to 0.20% (62% reduction), 1.50% (60% reduction), and 0.35% (61% reduction), respectively. With bilateral sensor fusion, a more generic classifier (i.e., fewer control restrictions) can match the state-of-the-art.

### Related work

Our protocol was nearly identical to Young et al. (2014a) and Spanias et al. (2015) but involved able-bodied subjects, no device, and wearable sensors. Using all ipsilateral sensors in this setup, we achieved average transitional error rates (5.94 ± 0.84%) that were roughly half those reported by Spanias et al. (2015) and Young et al. (2014a); steady-state error rates (1.25 ± 0.19%) were comparable. As expected, error rates were higher for transitions, which had fewer training examples and generally more variability. Although we used a more generic mode-specific classification scheme compared to Simon et al. (2017) by neither merging level walking and ramp ascent classes nor adding special classifier configurations (e.g., predict transitions between stairs and level ground during mid-swing or mid-stance), we achieved error rates approaching the state-of-the-art for unilateral-informed intent recognition control of a powered leg prosthesis. Because the addition of only one contralateral sensor (preferentially kinematic) significantly reduced error rates, our results suggest that substantial improvements in controllability may be achievable with minimal instrumentation of the contralateral limb. Unexpectedly, the error rate increased slightly instead of plateauing after the addition of four sensors. Although these consistent increases were not statistically significant, they suggest that the extraneous sensors may not only be redundant but also detrimental for intent recognition because they contribute to model overfitting and/or introduce undesirable sensor drift. From a practical standpoint, the total time required for instrumenting the subject would also be substantially reduced by using an optimized subset of sensors instead of all sensors. Using delayed transitions (i.e., windows starting 210 ms before the gait event and ending 90 ms after the gait event) has significantly reduced intent recognition errors for prosthesis control because delayed windows span both the onset of and continuation of movement (Simon et al., 2017). However, although offline error rates would likely have decreased further we chose not to implement this delay for the sake of clarity and generalizability to non-prosthesis applications for which such delays may not be a desirable tradeoff for accuracy.

We showed that unilateral (ipsilateral or contralateral) sensor sets performed comparably for single modalities, which had previously never been demonstrated for intent recognition control strategies. Thus, our results suggest that using signals from the non-affected leg (which alternates between serving as the leading and the trailing leg) for control and perhaps signals from the affected side for gait segmentation could be suitable for assisting individuals with severe unilateral impairment given proper training on how to perform transitions. Therefore, we believe high performance intent recognition control systems could still be realizable for a range of assistive devices with simple sensorization by integrating with wearable sensors and modifying a generic intent recognition control architecture. Our findings also showed that linear discriminant analysis (LDA) can perform as well as, if not better than, more complex algorithms such as support vector machines (SVM) and artificial neural networks (ANN) on a feature set with higher dimensionality (up to 22 sensors, 332 features) than we have previously used in an intent recognition framework for lower-limb prosthesis control (Spanias et al., 2015). To our knowledge, no other studies have used simultaneously recorded bilateral lower-limb neuromechanical signals from able-bodied individuals in an intent recognition control framework; therefore, these results for predicting locomotor activities for unimpaired individuals freely walking without a device also help to establish classification accuracy benchmarks for this framework.

Our offline study with an above-knee amputee subject also lays a foundation for hybrid setups combining device-embedded and wearable sensors by demonstrating the feasibility and success of using bilateral sensor fusion for intent recognition control of an assistive device for a clinical population. Although bilateral sensor fusion achieved modest reductions in offline error rate for the state-of-the-art classifier, the clinical significance of these improvements remains unknown without an online analysis with more subjects walking in settings more representative of home use (longer sessions, more variability in terrain, no clinician to check alignment or supervise, etc.). We also demonstrated that a more generic classifier using bilateral sensor fusion could match the state-of-the-art classifier, thus showing that bilateral sensor fusion presents an alternative set of tradeoffs (instrumenting the non-prosthesis side versus delayed transitions and no unique assistance mode for ramp ascent) for reducing error rates which may be preferable for some subjects. Aside from intent recognition, other applications of neuromechanical sensor fusion have included volitional control of ankle position for below-knee amputees (Au et al., 2005, 2008; Kannape and Herr, 2014) and stumble detection and classification (Zhang et al., 2011a). Beside neuromechanical sensor fusion, vision-based environmental sensing has also shown potential to improve control in an intent recognition framework (Zhang et al., 2011b; Krausz et al., 2015). Although we have focused on bilateral neuromechanical sensor fusion for intent recognition, there are many other potential applications of bilateral sensor fusion including measuring balance, controlling stumble recovery mechanisms, modulating a reference trajectory (e.g., estimating slope of an incline based on the leading leg), and estimating walking speed. There is also potential to use other modalities such as soft capacitive stretch sensors and vision in bilateral intent recognition systems.

## Limitations

The primary limitation of this study is that able-bodied subjects walked without an assistive device. Our setup represents the best case scenario of walking with a completely massless and transparent device; however, we believe this scenario is still valuable for establishing a device-agnostic upper bound for intent recognition. After showing the feasibility of intent recognition for a range spanning no gait impairment (able-bodied subjects without a device) to full impairment (amputee with a prosthesis), we believe that these strategies will reasonably generalize to individuals with an intermediate level of impairment who need some assistance from a powered orthosis or exoskeleton. The neuromechanical signals we collected came from wearable sensors only but capture information that aligns closely with control signals commonly accessible to wearable assistive devices. For example, the C-Leg and C-Brace typically use joint and/or limb kinematic information from sensors embedded in the device for control (Ottobock, 2015, 2016); in addition to these sources of information, the Vanderbilt leg has been controlled using signals from joint torque, load cell, and EMG sensors (Varol et al., 2010). Compared to sensors embedded in a device, wearable sensors are more susceptible to drift because they are not rigidly attached to the user; therefore, error rates are expected to decrease if the corresponding signals came from embedded sensors.

To be consistent with previous studies, our data collection used circuits consisting of level walking, ramps, and stairs only. This protocol is efficient but leads to the known issue of sparsity of transition examples, which were sometimes an order of magnitude less than steady-state steps for a given mode; however, we expect accuracy to increase with more data. Without load cell information, we chose an IMU-based segmentation approach which was not tuned for each subject's self-selected speed. Because the classifier relies on accurate and consistent detection of gait events, we confirmed that the segmentation algorithm's detection of gait events produced results that would have been similar to a thresholding approach based on axial load and joint kinematics as previously implemented on a powered knee-ankle prosthesis (Simon et al., 2014).

Another experimental limitation is that only relatively young, able-bodied subjects without any gait impairments participated in this study. We have included preliminary results from one above-knee amputee walking on a powered leg prosthesis but additional subjects (from different clinical populations and using different devices) are needed to establish the generalizability of a bilateral sensor fusion approach for intent recognition. Although the within-subject variability of control signals from an impaired population is expected to be higher, we still expect bilateral sensor sets to outperform their unilateral counterparts for individuals with unilateral impairment because the contralateral limb usually remains functional and likely a beneficial source of control information. Also, the results of evaluations done offline and with able-bodied subjects have generally been consistent with those from online testing (i.e., the user interacts with the device to control every step and can respond to errors) with amputee populations. Therefore, our promising results suggest that future efforts should be directed toward online testing with unilaterally-impaired individuals walking with an assistive device with some sensorization of the non-affected side to determine whether these significant improvements in offline accuracy translate to meaningful clinical benefit.

## Conclusion

We systematically demonstrated that using bilateral control signals consistently and significantly enhances offline accuracy for an intent recognition control system predicting locomotor activities. In particular, only one additional contralateral sensor was needed to provide significant benefit. Our work also establishes a benchmark for using bilateral lower-limb neuromechanical signals in a device-agnostic intent recognition control framework. We also provided preliminary evidence from an offline analysis with one above-knee amputee subject walking with a powered leg prosthesis to demonstrate the feasibility and benefit of integrating wearable sensors on the non-affected side to improve control using intent recognition. Together, these promising results also suggest that the intent recognition framework is compatible with a wide variety of sensor configurations and has potential to improve the control of many types of assistive devices.

## Author contributions

BH helped in conceiving the study concept, collecting, analyzing, and interpreting the data, and drafting the manuscript. ER helped in conceiving the study concept and interpreting the data, critically revising the manuscript for important intellectual content, obtaining funding, and supervising the study. LH helped in conceiving the study concept and interpreting the data, critically revising the manuscript for important intellectual content, obtaining funding, and supervising the study. All authors read and approved the final manuscript.

### Conflict of interest statement

The authors declare that the research was conducted in the absence of any commercial or financial relationships that could be construed as a potential conflict of interest. The reviewers JM and FOB and handling Editor declared their shared affiliation.
